# The Impact of Childhood Trauma on Fatigue: Preliminary Results Highlighting Emotion Dysregulation as a Mediator in Multiple Sclerosis

**DOI:** 10.1192/j.eurpsy.2025.854

**Published:** 2025-08-26

**Authors:** A. Süleyman, E. Akça, K. Ağan Yıldırım, G. Sünter, Ö. Yanartaş

**Affiliations:** 1 Psychiatry; 2 Neurology, Marmara University School of Medicine, Istanbul, Türkiye

## Abstract

**Introduction:**

Fatigue is one of the most significant factors impairing functionality in patients with multiple sclerosis (MS). Research has demonstrated that psychological factors, in addition to neurobiological ones, play a crucial role in fatigue among MS patients. Previous research has demonstrated that emotional neglect and emotional abuse are associated with fatigue in patients with MS (Pust et al. Front Psychiatry 2020; 11:811). While the role of emotion dysregulation as a mediator between adverse childhood experiences and long-term effects of childhood trauma has been studied, this relationship has not been previously examined in patients with MS.

**Objectives:**

This study aims to investigate the association between adverse childhood experiences and fatigue and to examine the mediating effect of emotion dysregulation in patients with MS.

**Methods:**

Patients with MS followed in the Neurology Outpatient Clinic at Marmara University, who were evaluated during their clinical examination to be cognitively competent and without any physical disabilities that would prevent them from completing the forms, were included in the study. Adverse childhood experiences were assessed using the expanded version of the Childhood Trauma Questionnaire (CTQ-33), emotion dysregulation was measured by the 16-item Difficulties in Emotion Regulation Scale (DERS-16) and fatigue was evaluated using the Fatigue Severity Scale. The impact of CTQ subscale scores on fatigue and the mediating role of emotion dysregulation were analyzed using SPSS with Process Macro v4.2.

**Results:**

A total of 119 patients completed the survey, with a mean age of 37.45 years, and 71.4% of the participants were women. Emotional abuse and emotional neglect were associated with fatigue in patients with MS. The effect of emotional abuse on fatigue was mediated by emotion dysregulation, with the total effect being 0.81 (95% CI [0.04, 1.58]), the direct effect -0.12 (95% CI [-0.86, 0.63]), and the indirect effect 0.93 (95% CI [0.50, 1.55]). Similarly, the relationship between emotional neglect and fatigue was also mediated by emotion dysregulation, with the total effect being 0.66 (95% CI [0.10, 1.22]), the direct effect -0.05 (95% CI [-0.60, 0.49]), and the indirect effect 0.71 (95% CI [0.39, 1.10]). These results indicate that emotion dysregulation fully mediates the effects of both emotional abuse and emotional neglect on fatigue in patients with MS.

**Conclusions:**

Our findings indicate that the link between fatigue severity and emotional neglect or abuse in MS patients is fully mediated by difficulties in emotion regulation. Consequently, it is suggested that interventions aimed at enhancing emotion regulation strategies may potentially mitigate the effects of adverse childhood experiences on MS-related fatigue. Further research, especially focused on intervention strategies, is needed in this area.

**Disclosure of Interest:**

None Declared

